# *De novo* sequencing, assembly and analysis of eight different transcriptomes from the Malayan pangolin

**DOI:** 10.1038/srep28199

**Published:** 2016-09-13

**Authors:** Aini Mohamed Yusoff, Tze King Tan, Ranjeev Hari, Klaus-Peter Koepfli, Wei Yee Wee, Agostinho Antunes, Frankie Thomas Sitam, Jeffrine Japning Rovie-Ryan, Kayal Vizi Karuppannan, Guat Jah Wong, Leonard Lipovich, Wesley C. Warren, Stephen J. O’Brien, Siew Woh Choo

**Affiliations:** 1 Genome Informatics Research Laboratory, High Impact Research (HIR) Building, University of Malaya, 50603 Kuala Lumpur, Malaysia; 2 Department of Oral and Craniofacial Sciences, Faculty of Dentistry, University of Malaya, 50603 Kuala Lumpur, Malaysia; 3 National Zoological Park, Smithsonian Conservation Biology Institute, Washington, DC 20008, USA; 4 CIIMAR/CIMAR, Interdisciplinary Centre of Marine and Environmental Research, University of Porto, Rua dos Bragas, 177, 4050-123 Porto, Portugal; 5 Department of Biology, Faculty of Sciences, University of Porto, Rua do Campo Alegre, 4169-007 Porto, Portugal; 6 Ex-Situ Conservation Division, Department of Wildlife and National Parks (DWNP) Peninsular Malaysia, KM 10, Jalan Cheras, 56100 Kuala Lumpur, Malaysia; 7 Center for Molecular Medicine and Genetics, Wayne State University, Detroit, MI 48201, USA; 8 Department of Neurology, School of Medicine, Wayne State University, Detroit, MI 48201, USA; 9 McDonnell Genome Institute, Washington University, St Louis, MO 63108, USA; 10 Theodosius Dobzhansky Center for Genome Bioinformatics St. Petersburg State University St. Petersburg, 199004, Russia; 11 Oceanographic Center, 8000 N. Ocean Drive, Nova Southeastern University, Ft Lauderdale, Florida 33004, USA

## Abstract

Pangolins are scale-covered mammals, containing eight endangered species. Maintaining pangolins in captivity is a significant challenge, in part because little is known about their genetics. Here we provide the first large-scale sequencing of the critically endangered *Manis javanica* transcriptomes from eight different organs using Illumina HiSeq technology, yielding ~75 Giga bases and 89,754 unigenes. We found some unigenes involved in the insect hormone biosynthesis pathway and also 747 lipids metabolism-related unigenes that may be insightful to understand the lipid metabolism system in pangolins. Comparative analysis between *M. javanica* and other mammals revealed many pangolin-specific genes significantly over-represented in stress-related processes, cell proliferation and external stimulus, probably reflecting the traits and adaptations of the analyzed pregnant female *M. javanica*. Our study provides an invaluable resource for future functional works that may be highly relevant for the conservation of pangolins.

The Malayan pangolin (*Manis javanica*) is a remarkable and distinctive mammal species belonging the order Pholidota, a sister taxon of Carnivora within the superclade Laurasiatheria. Pholidota pangolins are not closely related to other anteaters in South America (Xenarthra) although there are several morphological and adaptive similarities, some due to convergence but others likely inherited from the insectivore precursors of placental mammals that coexisted among the dinosaurs some 100MY ago^1^,^2^,^3^,^4^. Also being referred to as ‘scaly or spiny anteaters’, pangolins have a peculiar anatomy with keratinized scales made up of agglutinated hairs that overlap with one another and covering most of their body. Pangolins also lack teeth in adults (edentulism), an adaptation for the specialized diet made up of ants and termites (insect-eating - myrmecophagy)^1^ and possess ‘incomplete zygomatic arches’ and a ‘extremely reduced, bladelike mandible’ with each denture having a single bony protrusion^5^,^6^.

The number of Malayan pangolins in the wild is dramatically declining for several reasons and the species is characterized as being critically endangered in The International Union for Conservation of Nature and Natural Resources (IUCN) Red List of Threatened Species^7^,^8^. One of the major threats for its declining numbers is the rapid loss and deterioration of their natural habitat due to deforestation activities and human agricultural expansion^8^,^9^. Malayan pangolins (and pangolins in general) are also heavily hunted for their meat, skin, and scales, as illegal trade in live animals has become a severe threat to pangolins^10^,^11^,^12^. In China, pangolin meat is consumed as an exotic delicacy while the scales are used for traditional medicinal purposes such as skin diseases and cancer remedies, among other illnesses^8^. There has also been an attempt to relocate and breed the species in captivity while imitating its natural habitat, but with little success as pangolins do not survive and breed well in captivity^13^.

Genetic studies of endangered species have become widespread during the last several decades but more recently, the genomes and transcriptomes of endangered species have been sequenced^14^,^15^,^16^. With the emergence of high-throughput Next-Generation Sequencing (NGS) technologies, more molecular information about endangered species can be acquired and studied in-depth. Here, we present the sequencing of the first *M. javanica* transcriptomes, an important resource for the detailed study of this species that may assist the future management and conservation of this critically endangered mammal. We used short read Illumina HiSeq technology to sequence eight transcriptomes representing the following *M. javanica* organs: cerebellum, cerebrum, heart, kidney, liver, lung, spleen and thymus. The high quality transcriptomes generated were used for downstream analyses, which provided insights into functional and phylogenetic aspects of pangolin biology. The RNA-Seq reads are accessible at the Sequence Read Archive (SRA) at the National Center for Biotechnology Information (NCBI) under the accession number SRP064341. Genome raw reads and assemblies are also available for download at our repository http://pangolin-genome.um.edu.my or http://www.genomesolutions.com.my/iparc/pgd.

## Results

### RNA isolation and whole-transcriptome sequencing

To catalog a representative pangolin transcriptome, we sequenced eight RNA samples derived from a pregnant female *M. javanica* representing the following different organs provided by the Department of Wildlife and National Parks: cerebrum, cerebellum, thymus, liver, kidney, lung, spleen and heart. The quality of all extracted RNAs was evaluated using the Agilent Bioanalyzer 2100 (Agilent Technologies, Palo Alto, CA) before library preparation and sequencing. All samples had sufficient yields of RNAs with very good RNA integrity number (RIN) values (>9.00), indicating the high integrity of RNA samples for sequencing (Supplementary Figure 1). We generated approximately 373 million reads of 100 bp paired-end RNA-Seq data for the eight samples with the number of paired-end reads per sample ranging from 41,532,741 to 52,722,994 (Supplementary Table 1). The quality of sequencing reads was assessed based on the Phred scoring system^17^. More than 96% of reads for each organ had a read quality higher than the standard threshold (Q30), indicating good quality of the sequencing reads.

### Construction of the pangolin transcriptome

**Figure 1:**
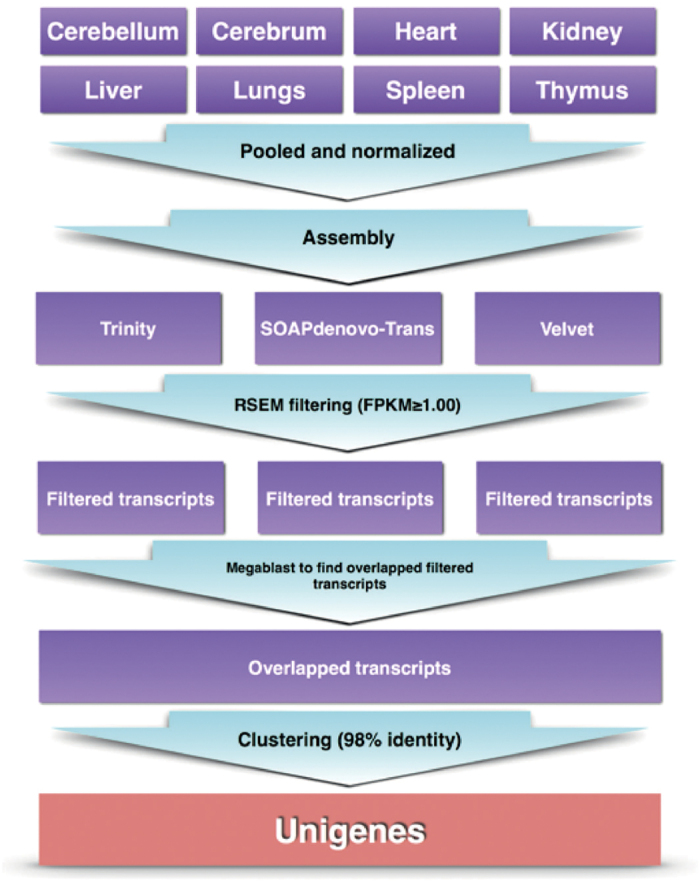
Assembly strategies to produce *M. javanica* transcriptome using three different assembly methods. The final *M. javanica* transcriptome was represented by unigenes, which are consensus assembled transcripts from three different assembly methods.

To generate a comprehensive and representative transcriptome of *M. javanica*, a total of 373,303,322 Illumina HiSeq reads were pooled from the eight different tissues. After normalization of *in silico* reads using a Perl script housed in the Trinity software package^18^, the reduced reads were assembled following the steps described in Fig. 1, using three different assemblers: Trinity^19^, SOAPdenovo-Trans^20^, and Velvet^21^. The consensus set of unigenes of the three different methods was used as the final representative transcriptome assembly, which resulted in 89,754 unigenes with a N50 of 3,741 bps (Supplementary Table 2). The length distribution of *M. javanica* assembled unigenes is displayed in Supplementary Figure 2 with comparison to the orthologous dog genes from ENSEMBL and the human genes from both ENSEMBL and RefSeq databases.

### Transcriptome assembly quality assessment

Although several pipelines to assess the quality of transcriptome assemblies have been recently developed and studied^22^,^23^, there are no established standard metrics for quantifying a transcriptome assembly, which is particularly difficult for a *de novo* non-model transcriptome assembly^24^. Therefore, we assessed the reconstructed *M. javanica* transcriptome using the following three *in silico* methods: (1) transcript sequence quality quantified by RNA-Seq by Expectation-Maximization (RSEM)^25^; (2) percentage of reads used to recover the unigene set as well as their coverage; and (3) sequence completeness and its contiguity.

All sequencing reads were filtered based on a Phred score of 30 and each assembled transcript was supported by a minimum Fragments per kilo base of transcript per million reads (FPKM) value of 1.00 (Supplementary Figure 3). When working with transcriptomes, read count is a way of quantifying the transcripts by abundance, and also determines whether or not the genes or transcripts produced are highly dependent on length and library size. Taking the FPKM values into consideration in generating the assemblies promotes normalization of the data, as well as minimizing the dependence of transcript selection based on the two variables, thereby producing high quality transcriptomes. RSEM outputs were used for transcript abundance estimation. Secondly, the normalized reads were mapped against the representative sequences. We observed that approximately 70% of the normalized paired reads were mapped back to the *M. javanica* unigenes with the mean mapping coverage per base of 27. The relatively high percentage indicates that a high amount of transcripts were recovered and retained for analyses after undergoing several stringent filtering steps.

Similarity search by Basic Local Alignment Search Tool (BLAST) against RefSeq database showed that 52.5% of *M. javanica* unigenes had significant matches (e-value <1e^−6^) with human and 40.1% with mouse. These results showed that most of *M. javanica* unigenes are homologous to its closely related and well-annotated mammalian (human and mouse) known genes, supporting that these unigenes are well-assembled.

To examine the completeness of the *M. javanica* unigenes, we used the TransDecoder program^18^. Our results showed that 57,214 sequences (63.7%) were protein-coding unigenes, with 38,168 unigenes predicted to have complete coding sequences (CDSs). Furthermore, 4,859 sequences were 3′ partial CDS while 12,486 were 5′ partial CDS. Only 1,701 sequences were internal sequences, which were defined as unknown. These results indicate a relatively high level of completeness of *M. javanica* unigenes based on the observations that most mammals have 20,000~30,000 gene loci in their genome^26^,^27^,^28^,^29^ and assuming that this pangolin species did not experience any lineage-specific whole-genome duplication^30^.

### Conservation among mammals

To the best of our knowledge, the *M. javanica* transcriptome reported in this study is the first Pholidota transcriptome ever sequenced. Because there was no reference pangolin genome available at the start of our study we compared the *M. javanica* unigenes against the NCBI non-redundant nucleotide database containing sequences for major organisms (from microorganisms to vertebrates) using BLASTX. The total number of BLAST top hit sequences was 69,565 with 55,568 sequences assigned to known species. As anticipated, the top 20 known species with the most abundant BLAST top hits are all derived from mammals with *Homo sapiens* (15.1%) being the highest, followed by the *Equus caballus* (12.3%), *Ailuropoda melanoleuca* (12.1%), *Canis lupus* (10.4%), and *Sus scrofa* (7.4%), supporting the view that the list of the assembled pangolin genes are highly similar to known mammalian genes.

### Unigene validation

**Figure 2:**
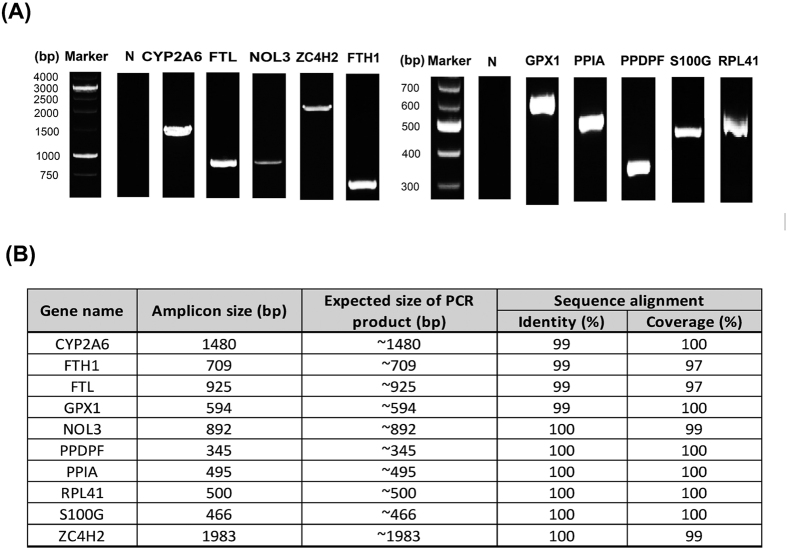
Validation of unigenes using PCR. The size of the PCR products of the validated unigenes meets well with the expected size. “N” is a negative control.

To further determine the quality and accuracy of *M. javanica* assembled unigenes, we performed Polymerase Chain Reaction (PCR) and Sanger sequencing in addition to *in silico* quality screening. A total of 10 unigenes were randomly selected for PCR and sequencing. The details of these sequences and the primer sequences used are described in Supplementary Table 3. Pooled RNA samples from the eight tissues were subjected to cDNA (complementary DNA) synthesis and PCR analysis. PCR products of the 10 genes matched the expected sizes (Fig. 2). To confirm the accuracy of each unigene, each PCR product was sequenced and its sequence was compared with the assembled unigenes. Our alignment results showed that the sequences from Sanger sequencing are almost perfectly aligned to our assembled unigenes with an average sequence identity of 99.6% and an average sequence completeness of 99.2% (Supplementary Table 3). These results further support the high quality of our *M. javanica* assembly.

### Gene Ontology (GO) and KEGG pathway analysis

To characterize the functional properties of the *M. javanica* transcriptome, unigene sequences were annotated using Blast2GO^31^. As a result, a total of 46,720 (52.1%) unigenes were assigned with GO terms (Supplementary Figure 5). As anticipated, functional annotation of *M. javanica* unigenes revealed high homology with known genes responsible for various biological roles. 50.4% of the unigenes were assigned to biological processes, 23.3% to molecular functions, and 26.3% to cellular components. For biological process, the most highly represented terms were cellular process (16.1%), metabolic process (14.2%) and single-organism process (13.1%). The fourth top represented term was biological regulation (12.0%), followed by response to stimulus (8.8%), cellular component organization (7.1%), developmental process (6.6%), signaling (6.4%), multicellular organismal process (5.9%), localization (5.4%), reproduction (2.1%), multi-organism process (1.1%), and growth (1.1%). The terms associated with reproduction, developmental processes, cellular component organization, and growth may be indicative of the involvement of the *M. javanica* transcriptome in various growth and developmental activities, which is common for an organism in a gestation period (the female pangolin used in this study was pregnant).

For molecular functions, the sequences were mainly assigned to binding (52.4%) and catalytic activity (25.9%), with the rest of the other terms distributed at 0.1–5.0%. As anticipated, cell (37.6%) and organelle (28.4%) are the most predominant terms assigned to the pangolin transcriptome in cellular components, followed by membrane (10.5%), macromolecular complex (10.2%), membrane-enclosed lumen (8.2%), extracellular region (4.1%), and lastly extracellular matrix (1.0%). Overall, these results are indicative of the broad range of biological activities related with the expressed pangolin transcriptome, representing a pooled collection of the multiple tissues sequenced.

**Figure 3:**
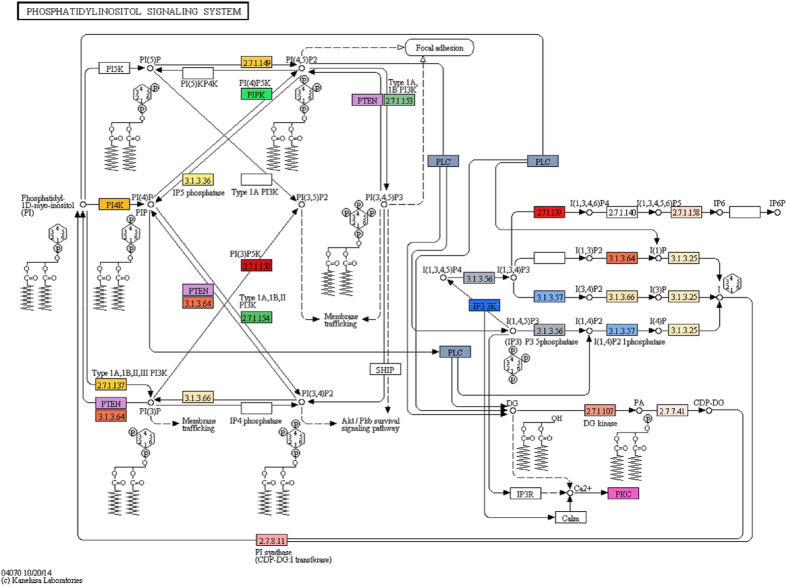
Phosphatidylinositol signaling pathway in the *M. javanica* transcriptome.

To identify the pathways in which the unigenes are involved, we mapped the unigenes of *M. javanica* on the known Kyoto Encyclopedia of Genes and Genomes (KEGG) pathways. We found a total of 19,165 (21.4%) *M. javanica* unigenes were associated with 138 unique KEGG pathways, with a total of 133 representing metabolic pathways, 3 environmental information processing pathways (signal transduction), 1 pathway in genetic information passing, and 1 pathway in organismal systems (immune systems) (Supplementary Table 4). The highest represented pathways in *M. javanica* unigenes included purine (2,784 unigenes) and thiamine (1,963 unigenes) metabolism, followed by aminobenzoate degradation (683), T cell receptor signaling pathway (582), and pyrimidine metabolism (489). For instance, we found that *M. javanica* unigenes were involved in the phosphatidylinositol signaling pathway (Fig. 3). Phosphatidylinositol 3-Kinase/Protein Kinase B signaling pathway (PI3K/Akt) involved in the phosphatidylinositol signaling pathway, is crucial for host survival (glycolysis/gluconeogenesis) and plays important roles in cell growth, proliferation (cell cycle) and survival signals (e.g. apoptosis)^32^,^33^. Interestingly, we also observed the involvement of *M. javanica* unigenes in the insect hormone biosynthesis pathway (KEGG map 00981). The genes that are associated with this pathway are MJU195075_c0_seq20, MJU195075_c0_seq3, MJU195075_c0_seq30, and MJU195075_c0_seq5. The biosynthesis of insect hormone belongs to the higher class of terpenoids and polyketides, and in vertebrates, is similar to cholesterol-driven pathway as shown in KEGG map 00981, which could be related to ant-eating traits. The future characterization of the pangolin digestive system transcriptome would be important to test the hypothesis that these genes are related to a specialized myrmecophagy phenotype^34^.

#### Lipid metabolism

Termites and insect larvae, a staple diet of pangolins, are rich in nutrition, especially fatty acids in the form of palmitic acid, oleic acid and linolenic acid^35^. In order to use them as an energy source, several important metabolic pathways are involved, namely the linoleic and alpha-linolenic acid metabolism pathways (KEGG:00591 & KEGG:00592), glycerolipid metabolism (KEGG:00561) and fatty acid degradation (KEGG:00071). A total of 747 unigenes were identified in the transcriptome that may participate in these pathways. These unigenes are involved in different pathways and some of them may overlap in different pathways (Supplementary Figure 6). These results provide an important resource in further understanding the potentially evolved lipid metabolism system of pangolins and improving their feeding management in captivity^36^. Likewise, the future characterization of the pangolin digestive transcriptome would be needed directly investigate the link between these lipid metabolism-related genes and the pangolin diet.

### Comparative analysis

**Figure 4:**
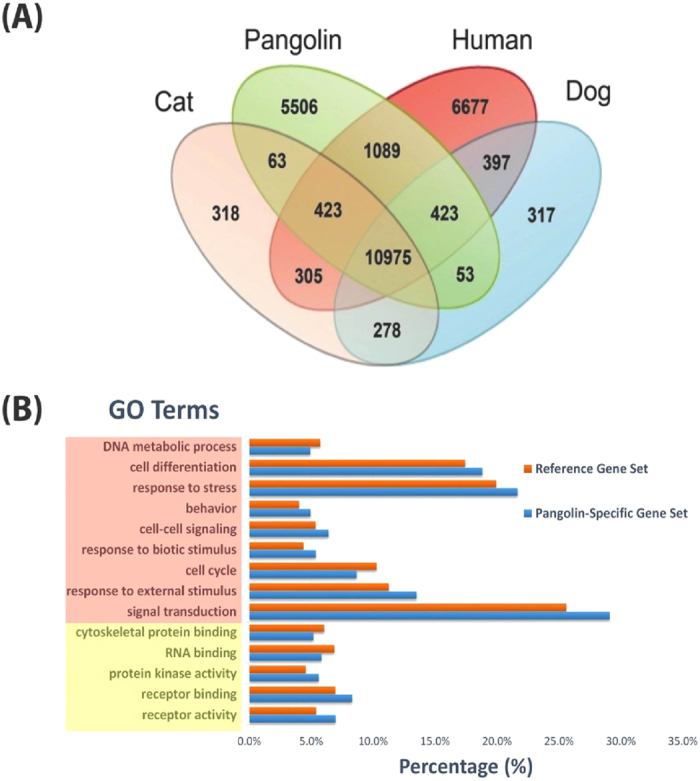
Comparative analysis. (**A**) Venn diagram showing comparison between the genes of pangolin and other mammals. (**B**) Functional enrichment analysis of pangolin-specific genes. Significant functional categories were shown. GO terms highlighted in light red are biological processes, whereas GO terms highlighted in yellow are molecular functions.

We compared pangolin unigenes with the well-annotated genes of *H. sapiens* and the closest relatives of pangolin from Carnivora, *Canis lupus familiaris* (dog) (CanFam 3.1, Ensembl Release 67) and *Felis catus* (cat) (Felis_catus-6.2, Ensembl Release 67) using OrthoMCL (Supplementary Table 5)^37^. We identified a large number of genes (5,506) specific to *M. javanica,* suggesting pangolins are divergent compared to their closest relatives (Fig. 4a). We used the Gene Ontology (GO) annotations to find the GO terms for which the *M. javanica* specific unigenes are enriched by running Fisher’s exact test (0.05 FDR) on a total of *M. javanica* specific unigenes. We identified nine significantly enriched GO terms in biological processes that include response to external stimulus, signal transduction, response to stress, cell proliferation, and response to biotic stimulus (Fig. 4b). Interestingly, most of these terms are well associated with cell interactions and response to stimulus, indicating that the *M. javanica* unigenes may be actively involved in response to stimulus or stress. At the molecular function level, the pangolin-specific genes are significantly enriched in functions such as cytoskeletal protein binding, receptor binding, receptor activity, RNA binding and protein kinase activity. Interestingly, the proteins involving in the cytoskeletal protein binding are known to interact selectively with any protein components of any cytoskeleton which includes actin, microtubule and intermediate filament cytoskeleton^38^,^39^. Pangolins have strong and sophisticated musculoskeletal system for digging and tree climbing^7^,^40^. Besides that, pangolins have a unique protecting mechanism allowing them to quickly roll themselves into visually impenetrable balls defending them against enemies, which may likely required the evolution of a sophisticated musculoskeletal system. Moreover, pangolin scales are structurally similar to human nails and hairs that are made of keratin^41^. The intermediate filaments could be transformed into keratin-like stretchable muscular-structure filaments, forming durable and strong scale structures. We hypothesize that the enriched pangolin-specific unigenes in cytoskeletal protein binding may be required for these unique features of pangolins (the development of pangolin keratinised scales and highly sophisticated musculoskeletal system for fossoriality or arboreality). However, further studies on these pangolin-specific genes may be required to insightfully understand the unique traits or adaptation of pangolins compared to other mammals.

### Repetitive elements discovery

Repetitive elements can usually be classified into two major categories: (1) transposon-derived interspersed repeats and (2) simple sequence repeats. These two repeat types can be further classified according to the pattern of repeating nucleotide base, mode of repeat expansion, and sequence homology with the consensus repeats family. These consensus repeat families are the core sequence from the repeat database RepBase, which is used by RepeatMasker^42^ to detect homologous repeats present in the transcripts. To identify the repeats in the *M. javanica* transcriptome, we screened the repetitive elements in the 89,754 unigenes using the RepeatMasker software^42^. For instance, we found many predicted repeats, covering a considerable portion (13.90% or 31,832,508 bp) of the total genomic length (229,081,942 bp) of all unigenes. The most represented repetitive elements were Long Interspersed Nuclear Elements (LINEs), where LINE1 family consists of 28,578 elements (4.67%), whereas Short Interspersed Elements (SINEs) have 33,009 elements (1.93%) in its Mammalian-wide interspersed repeats (MIRs) family (Table 1). The relatively high number of expressed repeats may be due to frequent exonization of transposable elements^43^ in pangolins. They may play an important role in pangolin transcriptome diversity as the repeats could introduce novel splice sites resulting in alternative splicing^44^.

Simple Sequence Repeats (SSR), also known as Short Tandem Repeats (STR) or microsatellites, are another major class of repetitive elements. Most of the SSRs found in the transcriptome or the protein-coding regions of the genome are trinucleotide repeats, since they do not cause frameshift mutations and retain protein integrity^45^. Trinucleotide repeats are known to affect the chemical and physical properties of certain proteins^46^ and length changes of triplet repeats are related to more than 40 neurological diseases found in humans^47^. The microsatellite searching tool MISA^48^ was used to find microsatellites present in the unigenes. In total, 45,223 Short Sequence Repeats (SSR) were identified from 89,754 non-redundant unigenes including their 5′- and 3′ UTR regions (Supplementary Table 6). Mono-nucleotides SSRs were the most abundant motif (20,705 copies), followed by di-nucleotide SSRs (20,064 repeats), tri- (4,001 repeats), tetra- (432 repeats) and penta-nucleotides (21 repeats) SSRs. The top mono-nucleotide repeat motifs and di-nucleotide repeat motifs included A/T (19,402 repeats or 42.9%) and AC/GT (10,776 repeats or 23.8%), respectively. Most of the SSRs found in the protein-coding regions of the genome are trinucleotide repeats (759 repeats). The major trinucleotide sequences found in the pangolin transcriptome coding regions are the AGG/CCT and CCG/CGG type. These putative SSRs could be useful as genetic markers for further investigations on genetic variation of the associated expressed genes.

### Pairwise comparisons of different transcriptomic profiles

**Figure 5:**
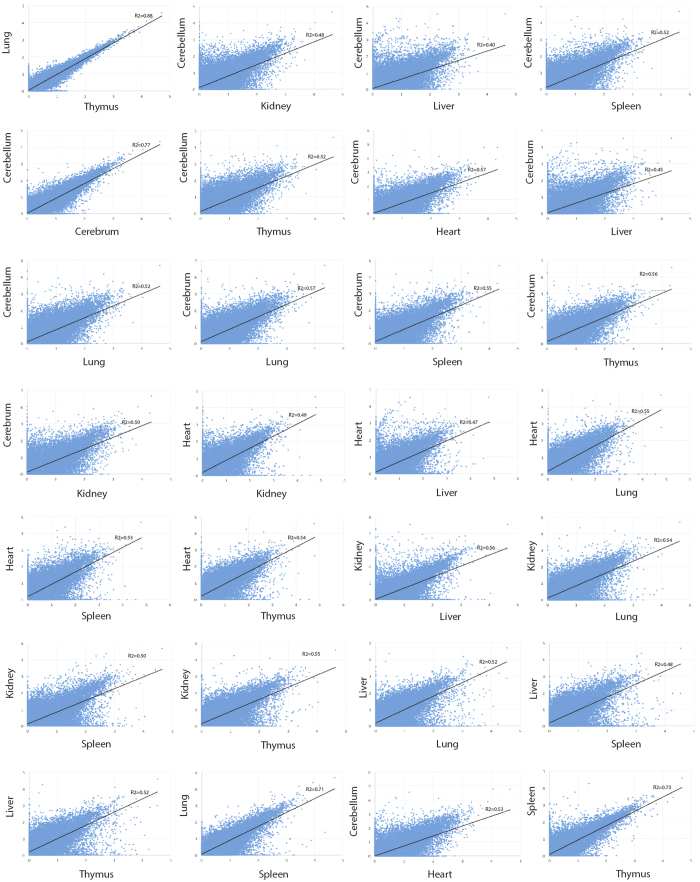
Correlation between any two organs resulting from pairwise comparison using expression values in log10 (FPKM+1) quantified by RSEM.

To examine the similarity of each organ transcriptome, we performed statistical correlation analysis for each pair of organs using log10 (FPKM + 1) to normalize the plots (Fig. 5). Our data showed that the thymus and lung transcriptomes have the most similar expression profiles (the coefficient of determination, R^2^ = 0.88), followed by cerebellum and cerebrum (R^2^ = 0.77). The cerebellum and liver have the least similar transcriptomic profiles with R^2^ = 0.40, reflecting the highly different complexity between the two organs^49^ As anticipated, the lung and thymus or the cerebellum and cerebrum showed the highest inter-tissue correlations, which is consistent with a previous study based on human tissues using gene expression profiles^50^. These results also fit into the biological origin of the tissues since they represent the immune system and brain, respectively.

### Discussion and conclusion

Pangolins are considered as one of the world’s most bizarre mammal. Belonging to the Pholidota, all eight species of extant pangolins are classified within the single family Manidae. Unfortunately, all the eight pangolin species are currently categorized from vulnerable to critically endangered, with *M. javanica* being one of the critically endangered species^7^. Many efforts have been made to maintain the population, but without success, in large part because pangolins do not survive and breed well in captivity^13^. Here we have successfully generated transcriptomic data from eight different organs of *M. javanica.* We have also assembled and reported the first comprehensive and representative catalog of *M. javanica* genes and their expression profiles across different organs as a starting platform to study the genomic and molecular basis of this lesser-known unique mammalian species.

Functional annotation of the *M. javanica* unigenes revealed the involvement of the species in various essential KEGG pathways such as T-cell receptor pathway and lipid metabolism. The lipid metabolism pathway, on the other hand, may support the myrmecophagus feeding habits of this mammalian species^1^,^35^.

Comparative analysis between *M. javanica* and its closest relatives, the Carnivora, revealed a large number of genes unique to pangolins (Fig. 4). These genes were significantly over-represented in biological processes such as response to external stimulus, response to stress and signal transduction. The enriched genes in these processes might be associated with the fact that the pangolin used here was pregnant. Another possible explanation is that the pangolin might be under stress after being caught or kept in captivity. Furthermore, we also cannot rule out the possibility that pangolins might have evolved to have different mechanisms to deal with stress and external stimulus compared to the closely related dogs and cats or even more distant humans. Studying and comparing the transcriptomes of more pangolins at different conditions may provide clearer insights into each possibility in the future.

In conclusion, we believe the high quality *M. javanica* transcriptome datasets serve as the first step towards understanding the uniquely specialized evolution of pangolins and a valuable genomic resource for functional studies on pangolins that may be important to unveil the mysteries of the biology and evolution of this rare and unique mammal.

## Methods

### Ethics statement

Veterinary officers conducted all procedures involving animals and experts at the Department of Wildlife and National Parks (DWNP), Malaysia, following internationally recognized guidelines and approved by the University of Malaya Institutional Animal Care and Use Committee (UM IACUC) [reference number of the approval: DRTU/11/10/2013/RH (R)].

### Biological sample

Briefly, a female pregnant pangolin sample weighing 2.73 kg was provided by the DWNP. Organ tissues (cerebellum, cerebrum, heart, kidney, liver, lungs, skin, spleen, and thymus) were harvested by DWNP veterinary officers and stored at −80 °C.

### RNA isolation and sequencing

Frozen tissues (~30 mg) were weighed and ruptured using TissueRuptor (Qiagen); followed by extraction using RNAeasy mini kit (Qiagen) following manufacturer’s manual. RNA concentrations were measured using Nanodrop^®^ ND-1000 Spectrophotometer (Thermo Scientific, Wilmington, DE). Quality of the isolated RNA was assessed using Agilent Bioanalyzer 2100 (Agilent Technologies, Palo Alto, CA), prior sequencing. Eight organs RNA sample (cerebellum, cerebrum, heart, kidney, liver, lungs, spleen, and thymus) of average insert size of 200 bps were sent for preprocessing. DNA contaminants were further removed using DNase enzyme digestion, followed by rRNA removal, then cDNA synthesis and PCR amplified into complete cDNA library sent for sequencing using Illumina HiSeq™ 2000 platform (2 × 100 bp strategy). The cDNA libraries were constructed according to Illumina TruSeq™ Stranded mRNA Sample Preparation Guide (Rev E, October 2013) for Illumina Paired-End Sequencing service provided by BGI, Hong Kong.

### *De novo* assembly strategies

Clean reads from Illumina HiSeq RNA-Seq were obtained after filtering out reads with adaptors, unknown nucleotides larger than 5%, and reads with low quality (more than 20% of the bases’ qualities are less than 10 in a read). A quality check was performed on the reads using the FastQC (version 0.10.1) software (http: //www.bioinformatics.bbsrc.ac.uk/projects/fastqc/).

*De novo* assembly of each individual tissue sample was performed using Trinity with default parameters previously described. To generate the whole transcriptome of Malayan pangolin, we assembled the pooled reads from eight samples using three different assemblers: Trinity^18^, SOAPdenovo-Trans^20^, and Velvet^21^. Total reads were first normalized by k-mer coverage using Trinity software. The assembled transcripts from three different assemblers were then individually filtered to remove poorly supported transcripts using the filtering criteria fragments per kilo base per million transcripts, FPKM, equals to 1.00.

Filtered transcripts from the three assemblers were then selected for overlaps and merged together; before being further clustered using CD-Hit-EST program^51^ using clustering threshold of 98% identity, to reduce redundancy. The singletons (unclustered transcripts), and the longest sequence representatives in each clustered transcripts were selected and retained, and classified as the unigenes; the sequences that cannot be extended on either ends. Figure 1 summarizes the workflow of *de novo* assembly to generate the Malayan pangolin unigenes. The unigenes then underwent foreign contamination screening (FCS) using BLAST with 98% identity cut-off to screen for any foreign organisms’ chromosome, mitochondria DNA, vectors, and sequencing adaptors.

### Functional annotation with GO and KEGG pathway analysis

To further validate and annotate the Malayan pangolin transcriptome, the assembled unigenes were subjected to sequence similarity search by BLASTX against NCBI’s non-redundant (nr) protein database with the configuration of E-value = 1e-3 and HSP length cut-off of 33. Results were exported into Blast2GO program to undergo mapping and functional annotation to retrieve GO terms associated with biological processes, cellular components, and molecular functions using the e-value hit-filter of 1e-6. Blast2GO also annotate the unigenes following KEGG database.

### Correlation between any two pangolin tissue transcriptomes

To examine the close-relatedness of pangolin tissue transcriptomes, the expression values of the unigenes (FPKM) in the transcriptomes of each tissue are manipulated. By utilizing the tool ‘RSEM-calculate-expression’ in the RSEM pipeline, the reads of each tissue were mapped to the unigenes^25^. Gene expression values, expressed as log_10_ (FPKM + 1) for each tissue transcriptome were plotted against one another producing the scatter plots. *R*^2^ values were then calculated from the scatter plots to estimate the correlation between any two pangolin transcriptomes.

## Additional Information

**How to cite this article**: Yusoff, A. M. *et al. De novo* sequencing, assembly and analysis of eight different transcriptomes from the Malayan pangolin. *Sci. Rep.* **6**, 28199; doi: 10.1038/srep28199 (2016).

## Supplementary Material

Supplementary Information

## Figures and Tables

**Table 1:** Repeat statistics in pangolin unigenes from RepeatMasker analysis.

Repeats Category	Repeats Family	Number of Elements	Length Occupied	Percentage of Sequence
SINEs	ALUs	0	0 bp	0%
	MIRs	33,009	4,412,161 bp	1.93%
	Total	33,304	4,448,145 bp	1.94%
LINEs	LINE1	28,578	10,701,435 bp	4.67%
	LINE2	19,964	4,376,160 bp	1.91%
	L3/CR1	2,163	387,385 bp	0.17%
	Total	51,456	15,616,512 bp	6.82%
LTR elements	ERVL	4,377	1,476,108 bp	0.64%
	ERVL-MaLRs	8,281	2,377,187 bp	1.04%
	ERV_classI	2,228	767,122 bp	0.33%
	ERV_classII	57	33,451 bp	0.01%
	Total	15,903	4,860,807 bp	2.12%
DNA elements	hAT-Charlie	15,550	2,780,742 bp	1.21%
	TcMar-Tigger	5,032	1,124,014 bp	0.49%
	Total	25,893	4,806,604 bp	2.10%
Unclassified:		237	37,076 bp	0.02%
Total interspersed repeats:		126,556	29,769,144 bp	12.99%
Small RNA:		328	40,193 bp	0.02%
Satellites:		63	8,316 bp	0%
Simple repeats:		38,549	1,676,576 bp	0.73%
Low complexity:		7,128	349,384 bp	0.15%
