# Supplementary material for: De novo sequencing, assembly and analysis of eight different transcriptomes from the Malayan pangolin
Source: Sci Rep. 2016 Sep 13;6:28199. doi: 10.1038/srep28199 (PMC5020319; doi:10.1038/srep28199)
Supplement: Supplementary Information [file srep28199-s1.pdf]

De novo sequencing, assembly and analysis of eight different transcriptomes from the Malayan pangolin

The International Pangolin Research Consortium (IPaRC)

Aini Mohamed Yusoff[1,4], Tze King Tan[1,4], Ranjeev Hari[1,4], Klaus-Peter Koepfli[7], Wei Yee Wee[1,4], Agostinho Antunes[8,9], Frankie Thomas Sitam[5], Jeffrine Rovie Ryan Japning[5], Kayal Vizi Karuppannan[5], Guat Jah Wong[1], Leonard Lipovich[10,11], Wesley C. Warren[3], Stephen J. O'Brien [2,6], Siew Woh Choo[1,4]

Affiliation addresses

[1]Genome Informatics Research Laboratory, High Impact Research (HIR) Building, University of Malaya, 50603 Kuala Lumpur, Malaysia.
[2] Theodosius Dobzhansky Center for Genome Bioinformatics St. Petersburg State University St. Petersburg, Russia 199004
[3]McDonnell Genome Institute, Washington University, St Louis, MO 63108, USA.
[4]Department of Oral Biology and Biomedical Sciences, Faculty of Dentistry, University of Malaya, 50603 Kuala Lumpur, Malaysia
[5]Ex-Situ Conservation Division, Department of Wildlife and National Parks, 10 Jalan Cheras, 56100 Kuala Lumpur, Malaysia.
[6]Oceanographic Center, 8000 N. Ocean Drive, Nova Southeastern University, Ft Lauderdale, Florida 33004, USA.
[7]National Zoological Park, Smithsonian Conservation Biology Institute, Washington, DC 20008, USA
[8]CIIMAR/CIMAR, Interdisciplinary Centre of Marine and Environmental Research, University of Porto, Rua dos Bragas, 177, 4050-123 Porto, Portugal.
[9]Department of Biology, Faculty of Sciences, University of Porto, Rua do Campo Alegre, 4169-007 Porto, Portugal.
[10]Center for Molecular Medicine and Genetics, Wayne State University, Detroit, MI 48201, USA.
[11]Department of Neurology, School of Medicine, Wayne State University, Detroit, MI 48201, USA.

Corresponding author:
Siew Woh Choo
lchoo@um.edu.my or l.choo@genomesolutions.com.my

Supplementary Figures

Supplementary Figure 1: Table of Bioanalyzer results indicating the quality of RNA extracted from respective tissue.

Organ/ details	Electropherogram
**Cerebellum**<br><br>RIN: 10.0<br><br>RNA area:196.6<br><br>RNA concentration: 113 ng/µl	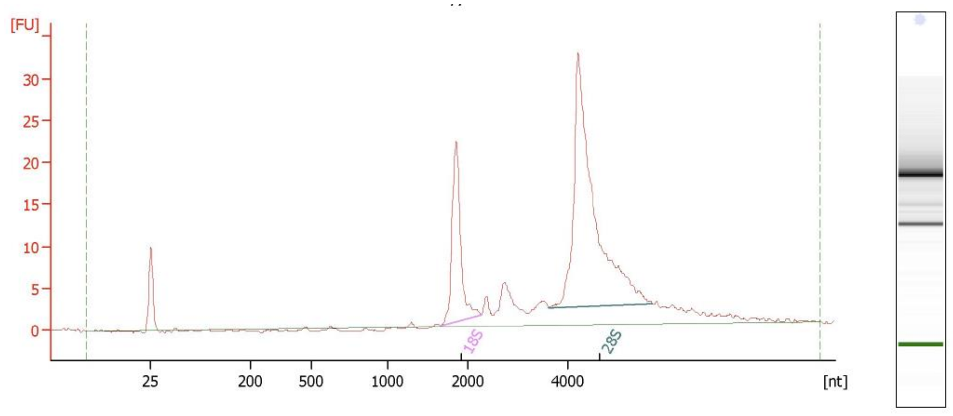
**Cerebrum**<br><br>RIN: 10.0<br><br>RNA area: 140.1<br><br>RNA concentration: 120 ng/µl	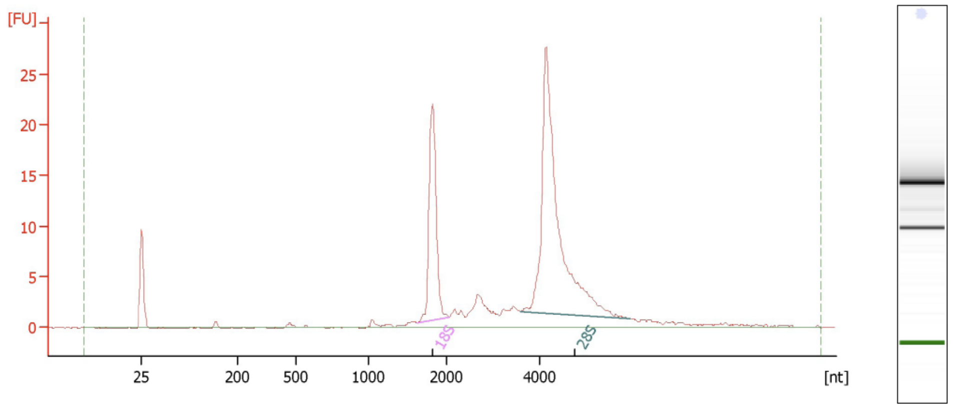
**Heart**<br><br>RIN: 9.30<br><br>RNA area:187.3<br><br>RNA concentration: 107 ng/µl	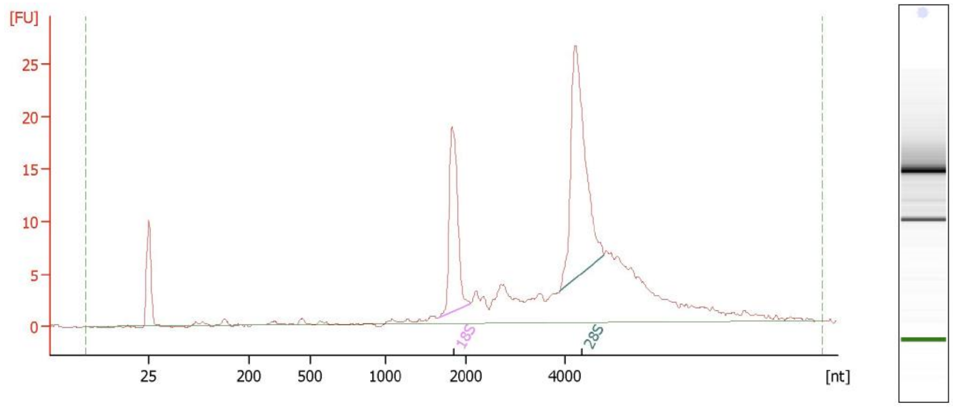

Organ/ details	Electropherogram
**Kidney**<br><br>RIN: 9.90<br><br>RNA area:639.6<br><br>RNA concentration: 550 ng/µl	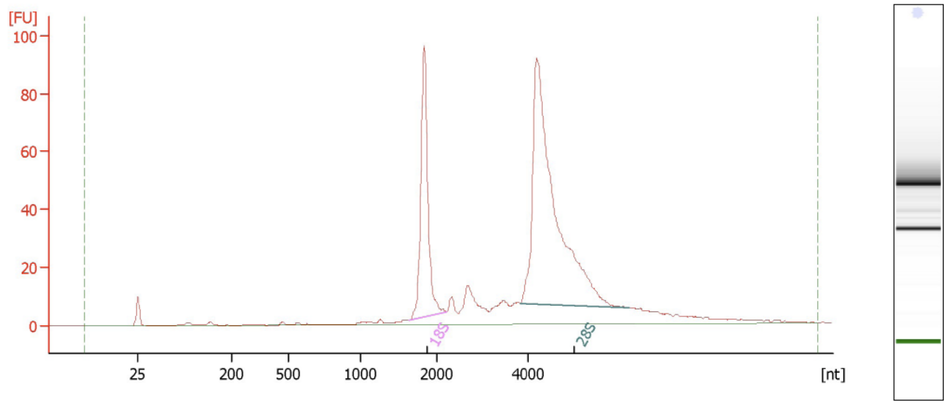
**Liver**<br><br>RIN: 9.10<br><br>RNA area:441.7<br><br>RNA concentration: 253 ng/µl	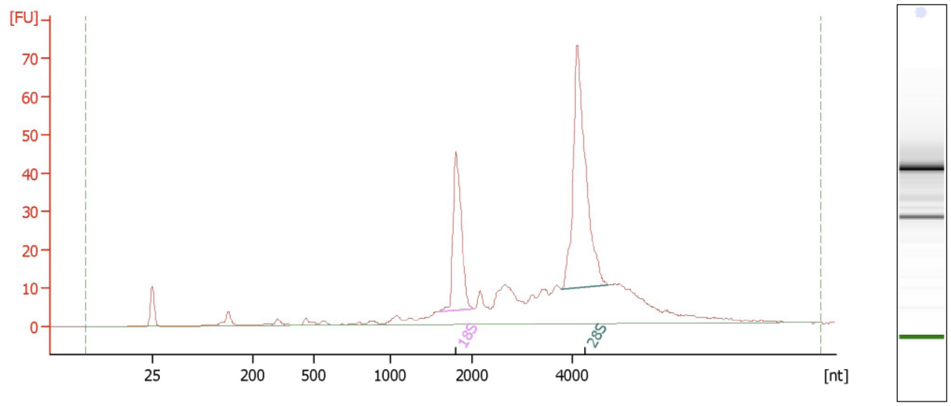
**Lungs**<br><br>RIN: 10.0<br><br>RNA area:324.7<br><br>RNA concentration: 279 ng/µl	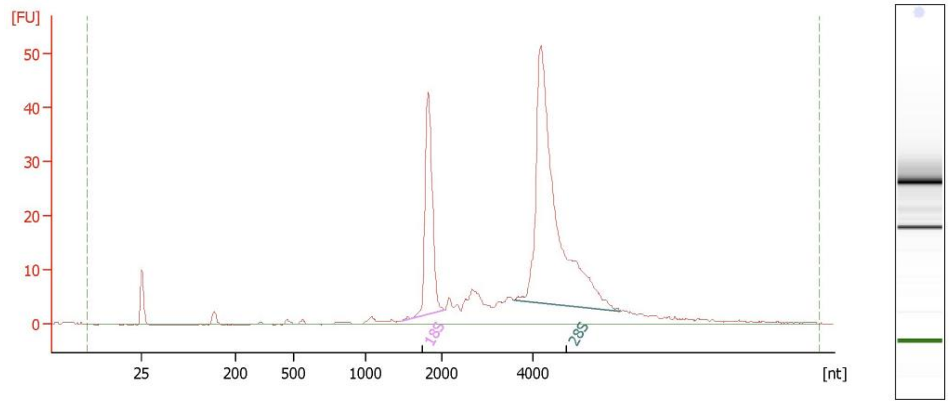

Organ/ details	Electropherogram
**Spleen**<br><br>RIN: 9.80<br><br>RNA area:544.0<br><br>RNA concentration: 312 ng/µl	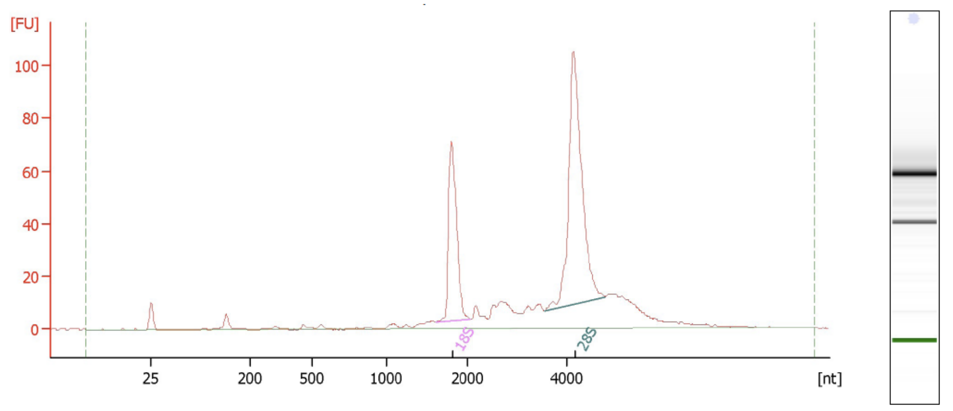
**Thymus**<br><br>RIN: 9.60<br><br>RNA area:236.9<br><br>RNA concentration: 204 ng/µl	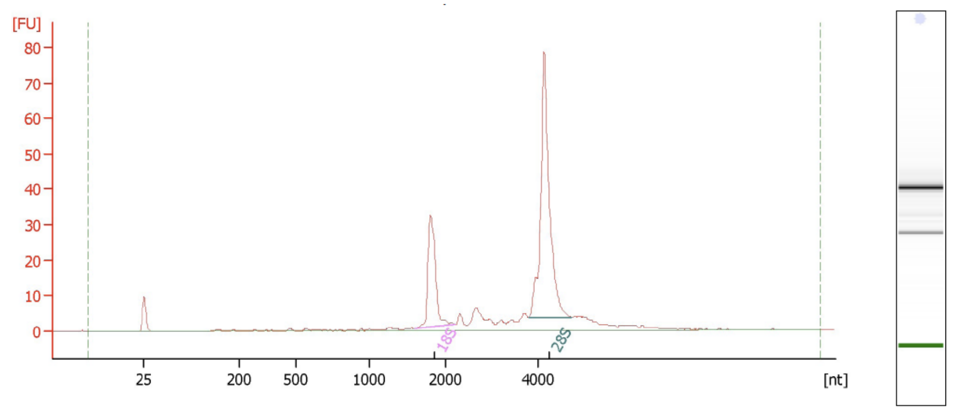

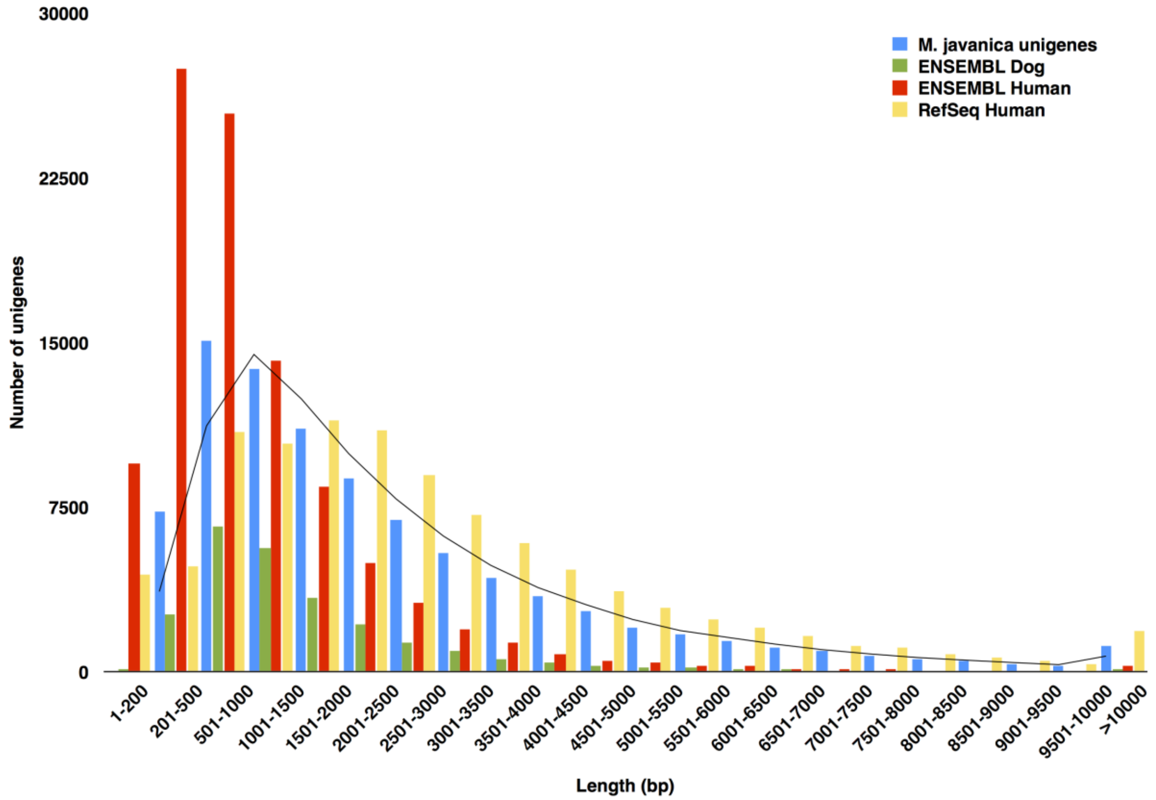

Supplementary Figure 2: *M. javanica* unigene length distribution in comparison with ENSEMBL dog and human genes, and RefSeq human genes.

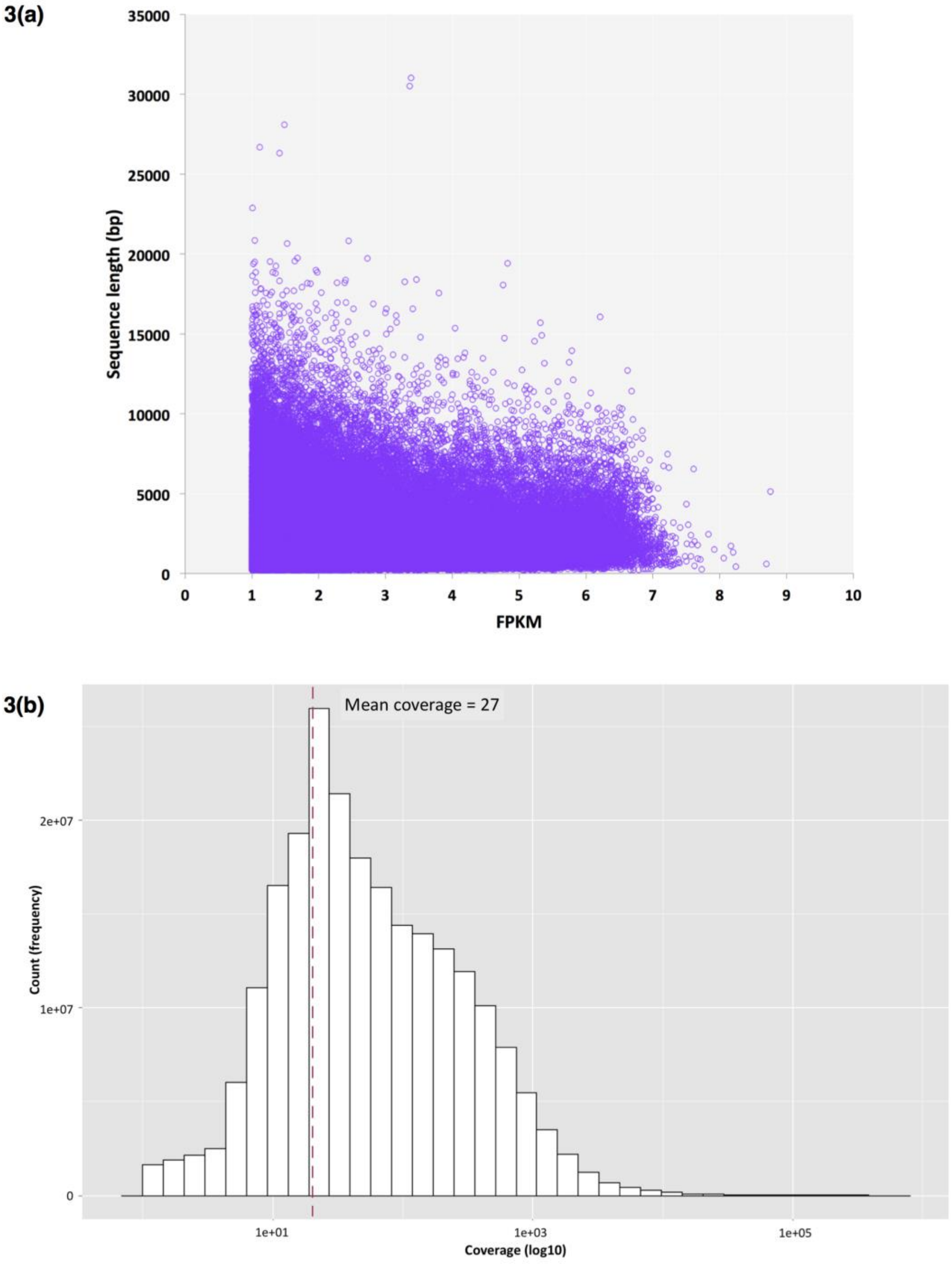

Supplementary Figure 3: *M. javanica* transcript validation and quality assessment. Figure 3a) describes the relationship between FPKM values and unigene length. The minimum supported FPKM is 1.00. Figure 3b) shows the mapping coverage distribution of M.javanica unigenes. The red dotted line indicates the mean mapping coverage, denoting the quality of mapping.

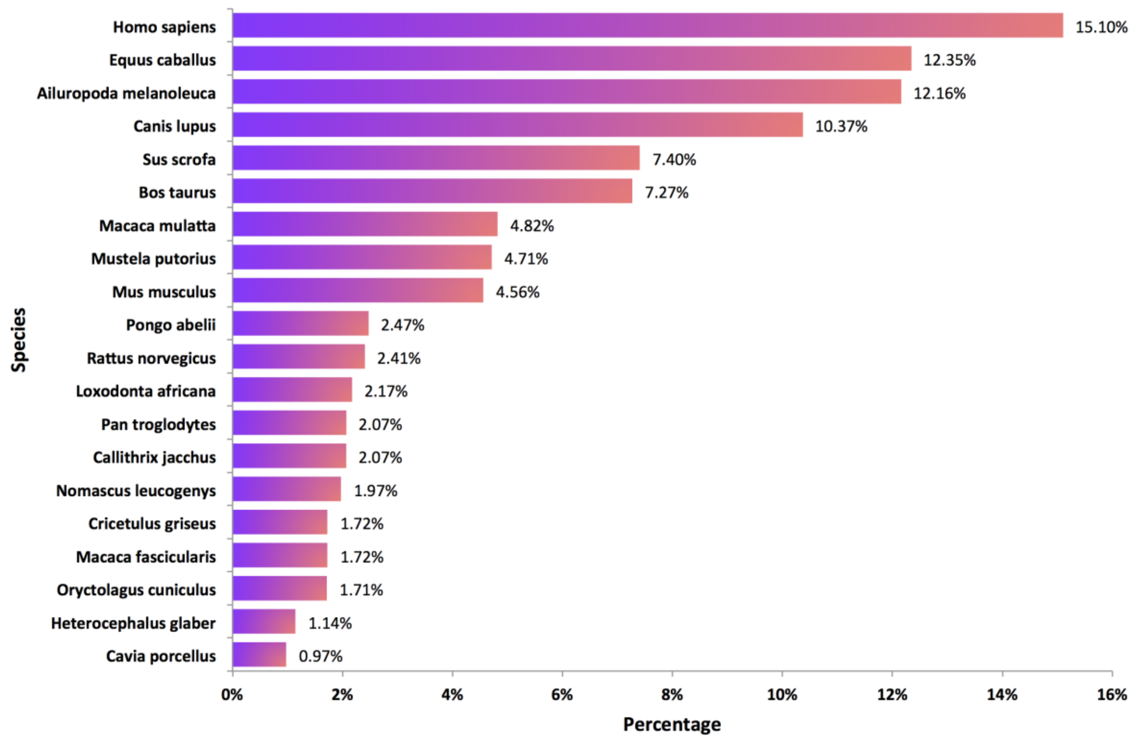

Supplementary Figure 4: Top 20 BLAST hits distribution to known species.

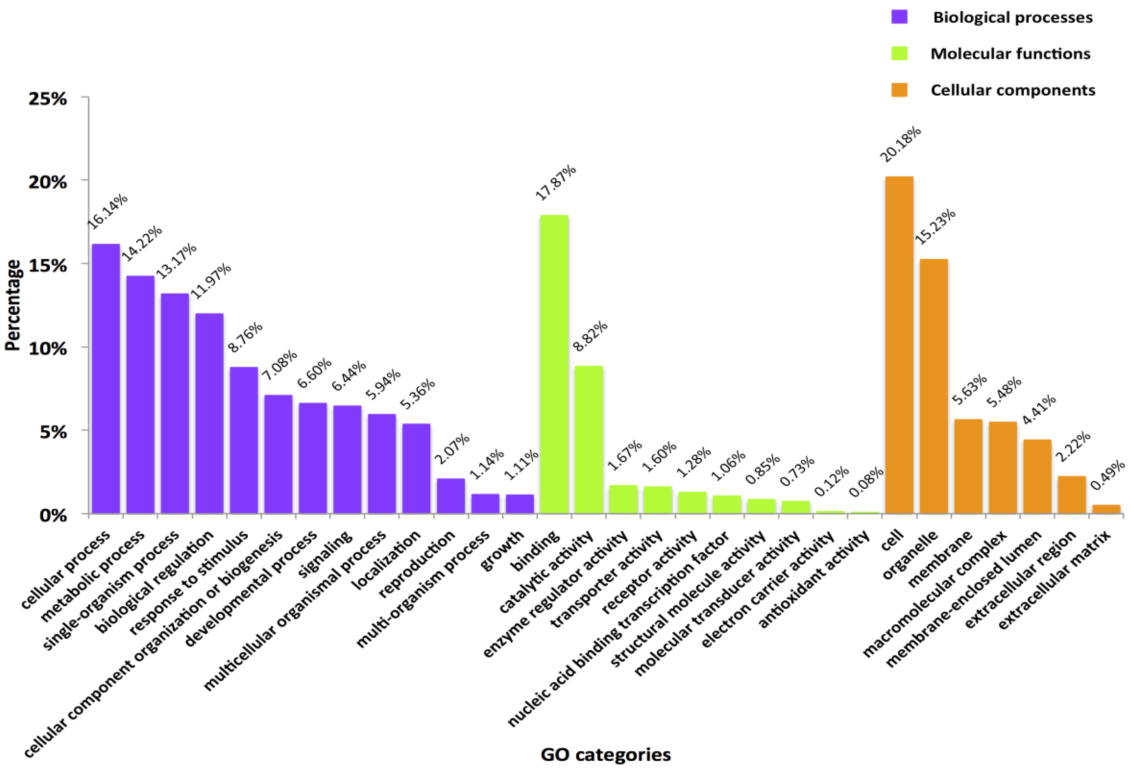

Supplementary Figure 5: Functional classification of assembled *M. javanica* unigenes.

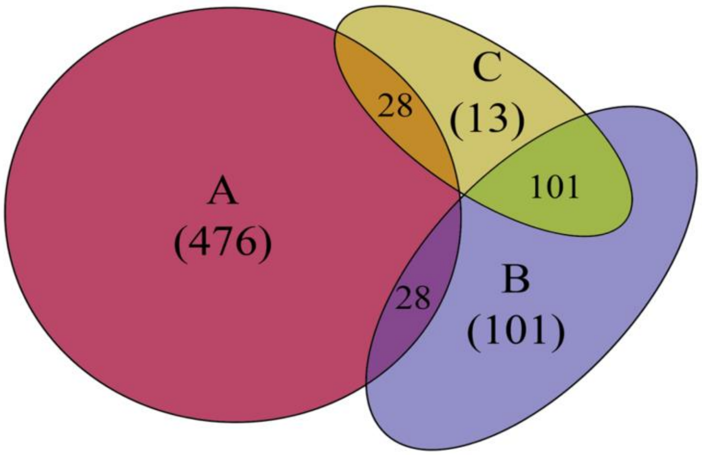

Supplementary Figure 6: Mapping of unigenes to metabolic pathways. A=Glycerolipid metabolism; B=Fatty acid degradation; and C=Linoleic and alpha-linolenic metabolism.
